# Inhibition of the NMDA receptor/Nitric Oxide pathway in the dorsolateral periaqueductal gray causes anxiolytic-like effects in rats submitted to the Vogel conflict test

**DOI:** 10.1186/1744-9081-5-40

**Published:** 2009-09-23

**Authors:** Lucas LM Tonetto, Ana L Terzian, Elaine A Del Bel, Francisco S Guimarães, Leonardo BM Resstel

**Affiliations:** 1 Department of Pharmacology, School of Medicine of Ribeirão Preto, University of São Paulo, Av Bandeirantes 3900, Ribeirão Preto, SP, 14049900, Brazil; 2 Department of MEF-Physiology, School of Odontology, University of São Paulo, Av do Café s/n, 14049900, Ribeirão Preto, SP, Brazil

## Abstract

**Background:**

Several studies had demonstrated the involvement of the dorsolateral portion of periaqueductal grey matter (dlPAG) in defensive responses. This region contains a significant number of neurons containing the enzyme nitric oxide synthase (NOS) and previous studies showed that non-selective NOS inhibition or glutamate NMDA-receptor antagonism in the dlPAG caused anxiolytic-like effects in the elevated plus maze.

**Methods:**

In the present study we verified if the NMDA/NO pathway in the dlPAG would also involve in the behavioral suppression observed in rats submitted to the Vogel conflict test. In addition, the involvement of this pathway was investigated by using a selective nNOS inhibitor, Nω-propyl-L-arginine (N-Propyl, 0.08 nmol/200 nL), a NO scavenger, carboxy-PTIO (c-PTIO, 2 nmol/200 nL) and a specific NMDA receptor antagonist, LY235959 (4 nmol/200 nL).

**Results:**

Intra-dlPAG microinjection of these drugs increased the number of punished licks without changing the number of unpunished licks or nociceptive threshold, as measure by the tail flick test.

**Conclusion:**

The results indicate that activation of NMDA receptors and increased production of NO in the dlPAG are involved in the anxiety behavior displayed by rats in the VCT.

## Background

The periaqueductal gray matter (PAG) is structure closely related to nociceptive and defensive responses which can be divided into four different columns along its rostro-caudal axis: dorsomedial, dorsolateral (dlPAG), lateral and ventrolateral columns [1-3]. Microinjection of NMDA receptor agonists into the dlPAG evokes flight reactions [4-6] and glutamate receptors are widely expressed in the dlPAG [7]. Moreover, antagonism of NMDA receptors AP7 in the dlPAG causes an anxiolytic-like effect in rats submitted to the elevated plus maze and Vogel conflict test [8-10]. These data indicate that glutamate receptors in the dlPAG play an important role in defensive responses.

Glutamate-NMDA receptor activation can induce the production of nitric oxide (NO) by activation of a calmodulin-dependent enzyme, the neuronal isoform of the nitric oxide synthase (nNOS), present in several corticolimbic structures [11,12]. nNOS inhibition promotes effects similar to those observed after NMDA antagonism [13,14]. Likewise, injections of non-selective NOS inhibitors into the dlPAG induced an anxiolytic-like effect in the elevated plus maze (EPM) model [15]. This model measures the conflict generated by the drive to explore a safe (closed arms) versus unsafe (open arms) place and is based on the innate fear of open spaces displayed by rodents. However, several pieces of evidence indicate that different aversive contingencies may engage distinct neurobiological systems [16]. It is important, though, to investigate drug effects in a specific brain site using different tests of anxiety.

The Vogel conflict test (VCT) is another animal model of anxiety that measures suppression of punished responses [16-22]. Different from the EPM, however, it is based on conflict induced by a learned contingency (electrical shock on a drinking spout) in thirsty animals [16,22].

Since the possible anxiolytic effects of NOS inhibition in the dlPAG have only been tested so far using the EPM, the aim of this study was to verify if the NMDA/NO pathway in the dlPAG is also involved in the behavioral suppression observed in rats submitted to the VCT. In addition, instead of the non-specific NOS inhibitors used in a previous work [15] we investigated the effects of a selective nNOS inhibitor, Nω-propyl-L-arginine (N-Propyl), and a NO scavenger, carboxy-PTIO (c-PTIO) microinjected into the dlPAG. These effects were compared to those produced by a specific NMDA receptor antagonist LY235959.

## Methods

### Subjects

Male Wistar rats weighing 230-250 g were used. Animals were kept in the Animal Care Unit of the Department of Pharmacology, School of Medicine of Ribeirão Preto, University of São Paulo. Rats were housed four per cage in plastic cages (18 x 32 x 40 cm) under standard laboratory conditions, with free access to food and water and under a 12 h light/dark cycle (lights on at 06:30 h). The Institution's housing conditions and the experimental procedures were previously approved by the local Animal Ethics Committee (process number: 067-2009).

Seven days before the experiments the rats were anesthetized with tribromoethanol (10 ml/kg i.p.) and fixed on a stereotaxic apparatus (Stoelting, Wood Dale, Illinois, USA). Immediately before the surgery they received antibiotic (0.2 ml/animal, I.M., Pentabiotico^®^, Fort Dodge) and anti-inflammatory (2.5 mg/Kg, s.c., Banamine^®^, Schering-Plough) treatment. After local anesthesia of the scalp with 2% lidocaine chloridrate with norepinephrine (Xylestesin^®^, Cristália), the skull was surgically exposed and stainless steel guide cannulae (0.6 mm OD) were unilaterally implanted on the right side aimed at the dorsolateral periaqueductal gray (coordinates: AP = 0 from lambda, L = 1.9 mm at an angle of 16°, D = 4.0 mm) [23] with the help of a stereotaxic apparatus. The cannula tip was located 1 mm dorsal to the final injection site. The cannulae were fixed to the skull with dental cement and one metal screw. An obturator inside the guide cannula prevented obstruction.

### Drugs

LY235959 (Tocris), Nω-propyl-L-arginine (Tocris, Ellisvalle, MO, USA) and carboxy-PTIO (S)-3-Carboxy-4-hydroxyphenylglicine (c-PTIO, RBI, St. Louis, MO, USA); were dissolved in sterile artificial cerebrospinal fluid (composition: NaCl 100 mM, Na_3_PO_4 _2 mM, KCl 2.5 mM, MgCl_2 _1 mM, NaHCO_3 _27 mM, CaCl_2 _2.5 mM; pH = 7.4).); tribromoethanol (Aldrich, St. Louis, MO, USA), and urethane (Sigma, St. Louis, MO, USA) were dissolved in saline.

#### Apparatus

##### Vogel conflict test

The Vogel conflict test was performed in a Plexiglas box (42 × 50 × 25 cm) with a stainless steel grid floor. The metallic spout of a drinking bottle containing water projected into the box. The contact of the animal with the spout and the grid floor closed an electrical circuit controlled by a sensor (Anxio-Meter model 102, Columbus, USA), which produced 7 pulses/s whenever the animal was in contact with both components. Each pulse was considered as a lick and at every 20 licks the animal received a 0.5 mA electrical shock on the metallic drinking spout for 2 s. The sensor recorded the total number of licks and shocks delivered during the test period. The whole apparatus was located inside a sound-attenuated cage [24,25].

##### Water consummatory evaluation

The apparatus was the same used in the test above except that the electrical shock delivering system was render inoperative. The number of unpunished licks was measured after 24 h (pre-drug treatment) and 48 h of water deprivation. In the latter situation the measurement of water consumption was performed 10 min after intra-dlPAG microinjections of vehicle, LY235959, N-Propyl or c-PTIO.

##### Tail-flick test

The apparatus consisted of an acrylic platform with a nichrome wire coil (Insight Instruments. Brazil) maintained at room temperature (24-26°C). The rats were gently handled and had their tails laid across the coil. The coil temperature was then raised at 9°C/s by the passage of electric current. The system had a cut-off time of 6 s to prevent tissue damage when the coil temperature approached 80°C. The time to withdraw the tail was recorded as tail-flick latency. The electric current was calibrated to provoke this reflex within 2.5-3.5 s in non-treated animals [15,25].

##### Experimental design

In the Vogel conflict test the animals received microinjection of 200 nL of vehicle, 4 nmol of LY235959, 0.08 nmol of N-Propyl or 2 nmol of c-PTIO. Each animal was used only once and the doses were based on those employed in previous studies using intra-cerebral administration [14,26,27]. Intracerebral injections were performed with a thin dental 33G needle (Small Parts, Miami Lakes, FL, USA) introduced through the guide cannula until its tip was 1.0 mm below the cannula end. A volume of 0.2 μl was injected in 30 s using a syringe (Hamilton, USA) connected to an infusion pump (Kd Scientific, USA). In order to prevent reflux the guide cannula was left in place for 30 s after the end of each injection. A polyethylene catheter (PE 10) was interposed between the upper end of the dental needle and the syringe. Morphine hydrochloride [9,24] was used as a positive control in the tail-flick test (see bellow).

#### Procedure

##### The Vogel conflict test

The animals were water deprived for 48 h before the test. After the first 24 h of deprivation they were allowed to freely drink for 3 min in the test cage in order to find the drinking bottle spout. Some animals did not find the spout and were not included in the experiment. Twenty-four h later the animals received the microinjections and after 10 min were placed into the test box. The test period lasted for 3 min and the animals received a 0.5 mA shock every 20 licks. During this period the number of licks and shocks delivered were registered.

##### Water consumption test

This test was performed in independent groups of animals and the procedure was the same used in the Vogel conflict test except that the electric shock delivering system was render inoperative. After the first 24 h of deprivation they were allowed to freely drink for 3 min in the test cage. Twenty four h later the animals received the microinjections and were again allowed to drink for 3 min. The number of licks during these two periods was registered.

##### Tail-flick test

The tail-flick test was conducted in independent groups of animals receiving vehicle, morphine, LY235959, N-Propyl or c-PTIO. The heating was applied to a portion of the ventral surface of the tail located between 4 and 6 cm from its end. The tail-flick latency was measured at 5-min intervals until a stable baseline (BL) was obtained over three consecutive trials. The latency was measured again 30 s after drug administration and then at 10-min intervals for up to 40 min [22]. Morphine was administered systemically (i.p.) 10 min before the test. Vehicle, LY235959, N-Propyl or c-PTIO was microinjected into the dlPAG as described above.

##### Histology

**Figure 1:**
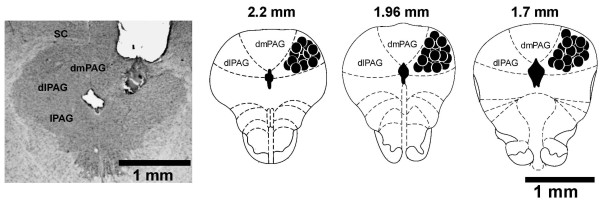
**Microinjections sites**. Photomicrograph of a coronal brain section showing an unilateral microinjection site in the dorsal periaqueductal gray and a histological localization of injection sites (200 nL) in diagrams based on the atlas of Paxinos and Watson [23]. The solid and the open circles represent injection sites inside of the dlPAG. Numbers represent distances from interaural level (mm). dPAG: dorsal periaqueductal gray; dlPAG: dorsolateral periaqueductal gray.

After the behavioral tests the rats were sacrificed under deep urethane anesthesia and perfused through the left ventricle of the heart with isotonic saline followed by 10% formalin solution. After that, the brains were removed and, after a minimum period of 5 days immersed in a 10% formalin solution, 50 μm sections were obtained in a Cryostat (Cryocut 1800). The injection sites were identified in diagrams from the Paxinos and Watson's atlas [23] and are illustrated in Figure 1. Rats receiving drug injections outside the dlPAG, dorsomedial PAG, lateral PAG and superior colliculus, were included in an additional (OUT) group.

### Statistical analysis

The data were expressed as mean ± S.E.M. The number of punished licks was analyzed by one-way ANOVA. The latency of tail withdrawal and water consumption were analyzed by a two-way repeated-measure ANOVA with treatment as the independent factor and time or day as the repeated measure. In case of significant interactions post-hoc comparisons were performed using the Dunnet's test. Results of statistical tests with P < 0.05 were considered significant.

## Results

### The Vogel conflict test

**Figure 2:**
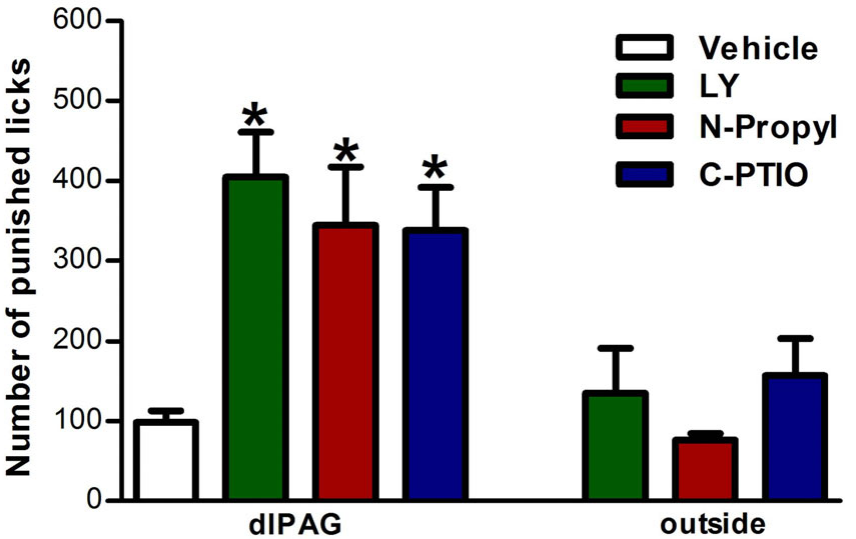
**Vogel conflict test**. Effects of LY235959 (4 nmol/200 nL), Nω-propyl-L-arginine (0.04 nmol/200 nL) and c-PTIO (1 nmol/200 nL) injected into the dlPAG of rats submitted to the Vogel test. Bars represent the mean ± SEM total number of punished licks in the 3 min session. Animals that received drugs outside the dlPAG were included as OUT groups. Asterisk indicates significant difference from vehicle (P < 0.05, ANOVA followed by the Dunnet's test; vehicle (veh, n = 10), LY235959 (n = 8), Nω-propyl-L-arginine (n = 7), c-PTIO (n = 9); OUT-LY235959 (n = 11), OUT-Nω-propyl-L-arginine (n = 5) and OUT-c-PTIO (n = 8).

In the first experiment, the animals that had received LY235959 (n = 8), N-propyl (n = 7) or c-PTIO (n = 9) into the dlPAG showed an increased the number of punished licks in the Vogel conflict test as compared to animals which had received vehicle (n = 10, F_6,57_= 6.71; P < 0.01, Figure 2). Moreover, drug injection outside this region (LY: n = 11, N-Propyl: 6 and c-PTIO: 8, Figure 2) did not modify the number of licks (P > 0.05).

### Drug effects on water consumption and tail-flick test

**Figure 3:**
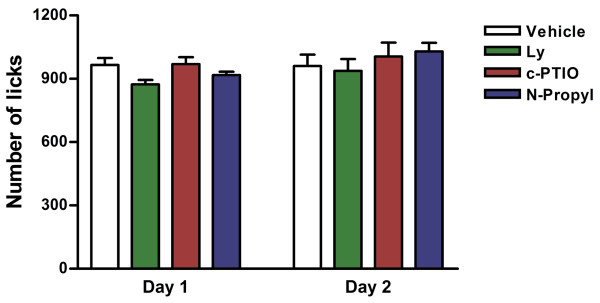
**Water consume test**. Effect of LY235959 (4 nmol/200 nL), Nω-propyl-L-arginine (0.04 nmol/200 nL) and c-PTIO (1 nmol/200 nL) injected into the dlPAG in the water consumption test during training (Day 1) and test (Day 2). Vehicle (veh, n = 6) LY235959 (n = 6), Nω-propyl-L-arginine (n = 6), c-PTIO (n = 6).

These additional experiments were performed to discard changes in nociceptive responses and water consumption as confounding factors in the Vogel test. In the water consumption test (Figure 3) neither drug (LY235959: n = 6, N-propyl: n = 6 and c-PTIO: n = 6) changed the number of unpunished lick compared to vehicle (n = 6, F_3,40 _= 1.380, P > 0.05). There was no difference for days (F_1,40 _= 2.8, P > 0.05), and no interaction treatment x drug (F_3,40 _= 0.63, P > 0.05).

**Figure 4:**
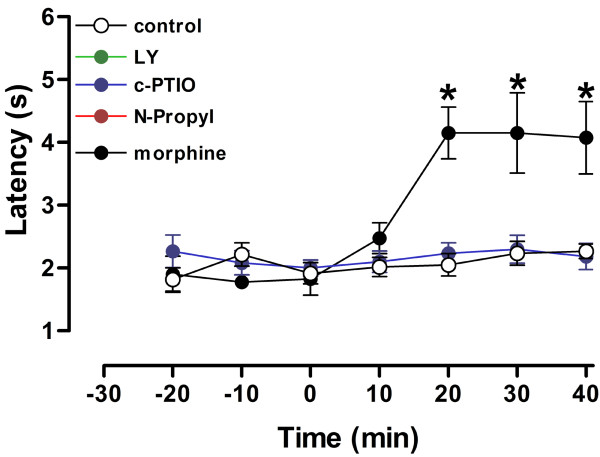
**Tail flick nociception test**. Time course of the effects of vehicle (veh, n = 6), LY235959 (n = 6), Nω-propyl-L-arginine (n = 6), c-PTIO (n = 6) or Morphine 5 mg/kg (n = 4) in the tail flick test. Each point represents the mean ± S.E.M. for the latency of tail withdrawal. Asterisk indicates significant difference from vehicle (P < 0.05, ANOVA followed by the Dunnet's test).

Finally, the tail-flick test results can be seen in Figure 4. There were a significant drug effect (LY235959: n = 6, N-propyl: n = 6 and c-PTIO: n = 6) (F_4,161 _= 14.59, P < 0.001), time (F_6,161 _= 9.65, P < 0.001) and drug × time interaction (F_24,161 _= 4.05, P < 0.001). Withdrawal latencies were significantly greater than vehicle at 20, 30 and 40 min after morphine injection (n = 5). No other difference against vehicle was found.

## Discussion

The present study showed that a selective nNOS inhibitor and a NO scavenger microinjected into the dlPAG produce effects similar to a glutamate NMDA receptor antagonist, increasing the number of punished licks in rats submitted to the VCT. The drugs did not interfere with the number of unpunished licks or nociceptive threshold measured in the tail flick test, indicating an anxiolytic-like effect.

The results confirm, using the highly selective NMDA-antagonist LY235959 [26,28,29], those previously obtained with AP7, another antagonist of these receptors, in the VCT and elevated plus maze [9]. They also agree with previous findings obtained after systemic injection of NMDA antagonists. For example, using systemic administration of noncompetitive or competitive NMDA receptor antagonists, Plaztnik et al. (1994) described increased punished responses induced by these drugs in the VCT. These antagonists also attenuated the anxious-like behavior observed in rats which had been previously exposed to a cat, suggesting that NMDA receptors are involved in the neural changes mediating the anxiogenic effect of severe stress [30]. Moreover, after repeated systemic administration in rats the NMDA receptor antagonist CGP 37849 not only retained its anxiolytic-like potency in the VCT but even enhanced rat exploratory behavior in a new environment, independently of changes in animal motor activity [31].

The present results reinforce the proposal that the dlPAG could be one of the brains sites where systemically administered NMDA-receptor antagonists exert their anxiolytic effects. The dlPAG is recognized as a key structure in the organization of defensive responses [3] and receives important projections from different cortical and limbic regions, including the prefrontal cortex and medial hypothalamus [1], that are intensively activated by anxiogenic drugs or by aversive stimuli [32,33]. Moreover, in addition to glutamate-receptors antagonists, anxiolytic effects have been observed after intra-dlPAG microinjection of drugs with different mechanisms of action, including benzodiazepines [34] and cannabinoids [24]. These latter drugs could be acting by inhibiting glutamate release [24,35].

In the central nervous system NO production by nNOS correlates with activation of NMDA receptors [36]. Moreover, NOS immunoreactive neurons are highly localized in the dlPAG [37]. Previous studies have shown that inhibition of NO formation or its effects in this region produces anxiolytic-like effects [13,15]. However, these studies employed non-selective NOS inhibitors and therefore could not exclude the involvement of other NOS isoforms. N-propyl is a NOS inhibitor that has a much higher potency to inhibit nNOS compared to eNOS or iNOS [38-40] whereas c-PTIO is a cell membrane-impermeable NO scavenger [41]. Together, the results indicate that that both nNOS activation and extracellular release of NO in the dlPAG are involved in anxiety modulation.

It has been showed by Aguiar *et al *[6] that that NO may have a facilitatory role in defensive reactions mediated by dlPAG. Corroborating our data, previous results had described that local NOS inhibition into the dlPAG, using an non-specific NOS inhibitor, induced anxiolytic-like effects in the elevated plus maze [15]. Our results using C-PTIO are in agreement with previous finds where C-PTIO injected into the dlPAG of rats exposed to the EPM evoked anxiolytic-like effect in this model [13,42]. Finally, the systemic administration the NOS inhibitor evoked anxiolytic like effects [43] and the benzodiazepine anxiolytic-like effect is NOS dependent in EPM [44].

Another caveat of the previous studies, as mentioned above, is that they were performed using only the EPM. It is important, however, to investigate the presence of anxiolytic drug effects in several animal models, since different aversive contingencies may engage distinct neurobiological systems [16]. In this way, although both the EPM and VCT are based on approach-avoidance conflict, the former relates to innate fear of open spaces against the drive to explore new environments whereas the latter involves fear of a learned contingency (electrical shock on a drinking spout) in thirsty animals [16,22,42].

## Conclusion

In summary, the present study provide evidence indicating that activation of NMDA receptors and increased production of NO in the dlPAG are involved in the anxiety behavior displayed by rats in the VCT.

## Financial competing interests

The authors declare that they have no competing interests.

## Authors' contributions

EADB, FSG, and LBMR contributed to the conception, design and critically revised the manuscript of the study. Moreover, LBMR was responsible by analysis and interpretation of data, drafted the manuscript and continuously supervised the study. LLMTand ALT contributed were responsible for data collection and helped to draft the manuscript. All authors read and approved the final manuscript.
